# Case of Obstinate and Fatal Vomiting, without Any Structural Disease of the Stomach

**Published:** 1818-10-01

**Authors:** M. Chomel


### *Case of obstinate and fatal Vomiting, without any Structural Disease of the Stomach.*

By M. Chomel.

In our last number we introduced a paper on *Morbid Anatomy*, from the pen of M. Bayle, in which was shewn the uncertainty that still attends the diagnosis between functional and structural derangements of internal organs; or, in other words, between idiopathic and sympathetic affections. Not a day passes without our witnessing examples of this uncertainty, in the confounding organic with functional diseases. The following case is highly instructive in many points of view, and therefore we shall be more minute than usual in its detail.

H. Franchebois, a female cook, 30 years of age, was received into the hospital La Charité, in the month of October, 1815. She had enjoyed good health, and led a regular life, up to January of the above year, when she began to feel a pain in the back part of the head, at first slight, but gradually augmenting in intensity, and stretching forward at last to the os frontis. Meantime the appetite and general strength began to decline; a diarrhœa and cough supervened, and she determined on going into hospital.

In about a fortnight after her entrance, the bowel complaint and cough ceased, but the disrelish for food continued, with pain in the epigastrium, and vomiting.

By the month of March, 1816, every kind of food was thrown up, except gum arabic and milk, which she occasionally retained on her stomach, and thenceforth formed her sole support. Various remedies were now tried, but without the least permanent effect.

In the month of May, 1816, our author examined this unfortunate young woman, and the following were the symptoms. A little to the right side of the epigastrium there was a *fixed pain*, which was augmented by inspiration or external pressure, with a sense of constriction always rendered more troublesome after taking food, of which, even in a liquid state, she could not take more than two or three ounces at a time. Immediately after swallowing the smallest quantity of her liquid food, she felt a sort of turning in the stomach, succeeded by a sense of weight, which lasted about a quarter of an hour; and if any liquid was taken before this sense of weight went off, it was sure to be vomited up. Eructations from the stomach were also frequent. Thirst constant; some pain about the umbilicus; borborigmi; obstinate constipation; being sometimes three weeks without a stool. Glysters produced such dreadful pain in the epigastric region, that she had seldom recourse to them but in the utmost extremity. The whole abdomen, when most carefully examined, presented no tumour or other tangible indication of organic disease. In other respects, the emaciation, which was but moderate on her entrance, had not increased; the complexion was clear; the physiognomy natural. She sat up a little every day, and slept about two hours each night. The head-ache now chiefly occupies the forehead. The respiration is easy, the pulse small, the skin soft, the urine pale; occasionally there are slight febrile movements in the system.

From this time till February 1817, there was little change. The vomiting and epigastric pain continued obstinate; the digestion became more and more difficult; the quantity of nourishment was obliged to be diminished, and was now only a few ounces of liquid in the day. The constipation was occasionally interrupted by a looseness in the bowels; the head-ache remained; the skin kept clear and soft; the emaciation did not advance.

During this long period, various means were tried; among which were cupping glasses to the region of the stomach; opium plasters to the same; fomentations; opium, musk, oxide of bismuth, &c. internally, but all in vain!

About the middle of February of this year, the pain of head became more violent; and on the 24th, the patient found herself extremely ill, and died in the evening, retaining her intellectual faculties to the last day.

Dissection. Notwithstanding the length of illness, and the extreme abstinence which she endured, the degree of emaciation was nothing remarkable. On the nates the fat was an inch in thickness! On opening the abdomen the *stomach* presented no lesion whatever, nor any thing uncommon either in colour or size. The same may be said of the *œsophagus*. The *lungs* contained a number of sparkling semi-transparent bodies of a line or two in diameter. The *brain* presented thirty or forty similar granulations, of a size and consistence resembling the chrystaline lens of the human eye. The *cerebellum* contained two of these bodies, and the spinal marrow one, opposite the last dorsal vertebra. In several points of the encephalon were seen small abscesses, about the same size as the forementioned pissiform bodies, and which appeared to be the same bodies in a state of suppuration.

In the *liver*, both on its surface and in its parenchymatous structure were discovered a great number of the same kind of bodies, as also in the spleen, kidnies, peritoneum, and diaphragm. The stomach and intestines presented none of these bodies. *Nouveau Journal de Medicine, March* 1818.

---

### REMARKS.

We may first observe, and indeed it is unequivocally acknowledged by the medical officers of La Charité, that, during the life of the patient, not an idea was entertained that the seat of the disease was any where but in the stomach; the organ *apparently* most affected. "*Nous n'avons pas soupçonné pendant la vie l'affection du cerveau; les vomissemens avaient, pour ainsi dire, absorbé toute notre attention.*" This was a grand oversight; and such as is too often committed among ourselves, where the *sympathetic* affections are not sufficiently attended to. The *symptoms* of the case in question were such, indeed, as would satisfy the superficial observer that there was organic disease in or near the stomach. But to those who have studied with care the phenomena of structural lesions of this and other interior organs, there would have appeared insuperable objections to the aforesaid conclusion. The pain in the head *preceding* and accompanying the gastric affection, was at least a suspicious circumstance. The non-correspondence of *emaciation* with the inability to take or retain food, was a very presumptive proof that the *structure* of the stomach was sound, however much its functions might be deranged. It is astonishing what a small supply of food will nourish the body, provided the digestive organs be uninjured in organization; but where there is organic lesion the body wastes rapidly, whatever the supply. The tint of the complexion, and the softness of the skin remaining natural, ought to have assured the medical attendants that the stomach was not materially implicated, except through sympathy. Lastly, abdominal *pressure* did not give that degree of pain which would have been felt, had organic disease existed in any part where pressure could be applied.

The case, however, offers a memorable example of sympathetic influence, and how far *functional* will imitate

*structural* disorders. It affords us, at the same time, some valuable *diagnostic* phænomena, distinguishing the two classes of disease, which it behoves the practitioner to bear constantly in mind. *Editor.*

